# Vaccination

**Published:** 1899-03

**Authors:** 


### VACCINATION.

All members of the medical profession and others who may have knowledge of injuries or sickness following upon vaccination are requested to communicate at once with C. Oscar Beasley, Esq., attorney, No. 311 Fidelity Building, Broad above Arch, Philadelphia, Pa.

Mr. Beasley is the attorney for the relator in the case of Field *vs.* McGlumphy, in which, for the first time in the history of vaccination, its real character has been put in issue in a court of justice, and the truth regarding it may now be judicially determined.

The case is one in which the constitutionality of the law of Pennsylvania, which requires children to be vaccinated as a condition of admission to the public schools, is contested on the express ground that vaccination is both useless and dangerous.

Approved by the Brooklyn (N. Y.) Anti-Compulsory Vaccination League, M. R. Leverson, President; and by the Anti-Vaccination Society of America.

In the above statement we have called attention to the case of Field *vs.* McGlumphy, brought in the Court of Common Pleas (Philadelphia), to test the validity of the vaccination laws of Pennsylvania.

We now invite all who can afford to do so to contribute towards the expenses of getting the case properly before the court. It will be readily understood that the amount of good work which can be done will be necessarily limited by the fund raised.

It will cost considerable to collect the necessary testimony, and it is important that, when collected, this testimony be printed, and this testimony is likely to become voluminous. There are also costly drawings to be prepared and laid before the court.

The whole expense of fighting vaccination in this country has hitherto been borne by a few persons of very moderate means, who have made very heavy sacrifices, both of time and money, to rescue the people from this, which Dr. Creighton, testifying before the recent Royal (British) Commission, called a "grotesque superstition," and which other pathologists term a murderous one.

The burthen of the present contest, which is likely to be a decisive battle, should not be thrown upon the few who have hitherto borne the brunt of the fight, and we urge all who care at all for the public welfare and the establishment of the truth, to contribute whatever they can afford to secure these ends.

Contributions may be sent to the publisher of this journal or to C. Oscar Beasley, Esq., attorney, 311 Fidelity Building, Broad above Arch, Philadelphia, Pa.

Approved by the Brooklyn (N. Y.) Anti-Compulsory Vaccination League, M. R. Leverson, President, and by the Anti-Vaccination Society of America.

